# Comparison of metabolic ratios of urinary estrogens between benign and malignant thyroid tumors in postmenopausal women

**DOI:** 10.1186/1472-6890-13-25

**Published:** 2013-10-25

**Authors:** Ju-Yeon Moon, Eun Jig Lee, Woong Youn Chung, Myeong Hee Moon, Bong Chul Chung, Man Ho Choi

**Affiliations:** 1 Future Convergence Research Division, Korea Institute of Science and Technology, 39–1 Hawolkok-dong, Seoul 136-791, Korea; 2 Department of Chemistry, Yonsei University, Seoul 120-749, Korea; 3 Department of Internal Medicine, Yonsei University College of Medicine, Seoul 120-752, Korea; 4 Department of Surgery, Yonsei University College of Medicine, Seoul 120-752, South Korea

**Keywords:** Estrogens, Postmenopause, Thyroid cancer, 16α-hydroxylation, 17β-hydroxysteroid dehydrogenase

## Abstract

**Background:**

Estrogen metabolism may be associated with the pathophysiological development of papillary thyroid carcinoma (PTC).

**Methods:**

To evaluate the differential estrogen metabolism between benign and malignant PTCs, estrogen profiling by gas chromatography–mass spectrometry was applied to urine samples from postmenopausal patients with 9 benign tumors and 18 malignant stage I and III/IV PTCs.

**Results:**

The urinary concentration of 2-methoxyestradiol was significantly lower in the stage I malignant patients (3.5-fold; *P* < 0.025) than in the benign group. The metabolic ratios of 16α-OH-estrone/estrone and estriol/estradiol, which are responsible for 16α-hydroxylase activity, were increased more than 2.5-fold in the advanced-stage malignant PTC (*P* < 0.02 each). The more than 6.2-fold decrease in the urinary 2-/16α-hydroxylase ratio in stage III/IV malignant PTC was consistent with the ratio in postmenopausal patients with endocrine gland cancers. In addition, reductive 17β-hydroxysteroid dehydrogenase (17β-HSD; estradiol/estrone or estriol/16α-OH-estrone) was present at significantly higher levels in subjects with stage III/IV malignant PTCs than in benign subjects (>3.5-fold difference; *P* < 0.002). In particular, the estriol/16α-OH-estrone ratio differentiated between the benign and early-stage malignant patients (*P* < 0.01).

**Conclusions:**

Increased 16α-hydroxylation and/or a decreased 2-/16α-ratio, as well increased reductive 17β-HSD, with regard to estrogen metabolism could provide potential biomarkers. The devised profiles could be useful for differentiating malignant thyroid carcinomas from benign adenomas in postmenopausal women.

## Background

Papillary thyroid carcinoma (PTC), the most frequently occurring thyroid carcinoma, is a well-differentiated endocrine-based tumor [1] with a 3-fold higher incidence in women than in men [2]. The identification of thyroid nodule malignancy is based on ultrasonography, scintigraphy, and fine-needle aspiration biopsy (FNAB) [3]. However, the results are limited to the differentiation of benign adenomas (adenomatous hyperplasia) from malignant thyroid carcinomas [4]. Because the prognosis is strongly determined by the tumor stage at diagnosis and the initial treatment, regardless of the patient age and histological type (especially in postmenopausal women) [5], novel and reliable biomarkers for early-stage diagnosis of thyroid malignancy that could noninvasively discriminate between malignant and benign nodules are necessary.

The identification of potential gene or protein expression markers for benign/malignant classifications was performed *in vitro* and *in vivo* using immunohistochemistry, Western blotting [6,7], or multi-gene approaches with microarray technology and molecular profiling of thyroid tissues from FNABs [8-10]. In recent, the pathophysiological roles of estrogen metabolism have been investigated with regard to human thyroid gland development [11-13], and estrogen metabolic alterations have been identified in urine samples from PTC patients, along with sex differences and menopausal conditions [14].

Estrogens are involved in the pathogenesis of thyroid nodules and differentiated thyroid cancer cells [15]. Benign and malignant thyroid cells and tissues express functional estrogen receptors (ERs), and their growth is stimulated by estrogen. In addition, estrogen can stimulate the growth and simultaneously inhibit the differentiation of thyroid nodule-derived stem/progenitor cells [16], which are adult stem cells that have been suggested as an alternative source of benign and malignant tumor formation [17]. There are 2 main estrogen metabolic pathways: the first is 16α-hydroxylation, which produces both estriol (16α-hydroxyestradiol) and 16α-hydroxyestrone, and the second is 2- and/or 4-hydroxylation to form 2- and 4-catechol estrogens [18,19]. In general, women with increased 16α-hydroxylation are thought to have an elevated risk of breast cancer, compared with women with increased 2-hydroxylation estrogen metabolism [20-23]. Thus, a decreased 2- to 16α-hydroxylation ratio has been shown to precede clinical evidence of cancer and therefore represents a significant risk factor for estrogen-dependent tumor development. However, the pathogenic differences in estrogen metabolism between benign and malignant thyroid tumors have not been fully explored, especially in postmenopausal women.

Gas chromatography–mass spectrometry (GC-MS)-based steroid profiling is a powerful technique that not only can describe risk associations and relationships between steroid hormones and clinical tumor growth characteristics but can also be used as a biomarker tool for discriminating benign from malignant adrenal tumors [24,25]. For low-level quantification, we developed a comprehensive urinary estrogen profile from osteoporotic postmenopausal women [26]. The aim of this study was to assess the differential expression of estrogen metabolites and their metabolic ratios between benign adenoma and malignant PTC patients to elucidate the underlying mechanisms of thyroid cancer pathology, as well as to identify potent biomarkers that are predictive of clinical characteristics. In addition, our results address the issue of whether preferential downregulation of the 2-/16α-hydroxylation ratio in postmenopausal women with thyroid malignancies could provide a useful presurgical classification for determining malignancy in thyroid carcinomas, as in other endocrine gland cancers.

## Methods

### Subjects and sample collection

Urine samples were collected from postmenopausal patients with a total of 18 malignant PTCs, including 11 women diagnosed with stage I (age: 56.1 ± 3.7 yr, body mass index [BMI]: 26.0 ± 3.3 kg/m^2^) and 7 women diagnosed with stage III/IV disease (age: 59.6 ± 10.9 yr, BMI: 22.9 ± 2.7 kg/m^2^) at Severance Hospital (Seoul, Korea). In addition, samples from 9 women diagnosed with benign tumors (adenomatous hyperplasia; age: 59.2 ± 5.8 yr, BMI: 26.5 ± 3.8 kg/m^2^) were included as a control group. All the subjects underwent the same diagnostic procedures, including a pathological examination, ultrasonography, and FNAB, as detailed by the American Joint Committee on Cancer staging criteria [3]. Patients with a history of cervical, breast, endometrial, or head and neck cancer and those with a history of respiratory papillomatosis were excluded. All the subjects had normal thyroid function (T3: 104.11 ± 19.1 ng/dL, T4: 11.16 ± 2.56 μg/dL, and TSH: 1.02 ± 0.31 μIU/mL) and were not treated with or exposed to any drugs or exogenous hormones for defined time periods. The experimental protocol (no. 4-2009-0424) was approved by the Institutional Review Board (IRB) Committee of the Human Research Protection Center at Severance Hospital, and informed written consent was obtained from all the subjects. All urine samples were collected in the morning after 12 h of fasting and immediately stored at -20°C until used. The urinary steroid levels were calibrated with creatinine values, according to Jaffé’s method [27].

### Chemicals and materials

Reference standards of the 16 estrogens, including estrone (E1), 17β-estradiol (E2), estriol (E3), 2-hydroxyestrone (2-OH-E1), 2-hydroxy-17β-estradiol (2-OH-E2), 4-hydroxyestrone (4-OH-E1), 4-hydroxy-17β-estradiol (4-OH-E2), 2-methoxyestrone (2-MeO-E1), 2-methoxy-17β-estradiol (2-MeO-E2), 4-methoxyestrone (4-MeO-E1), 4-methoxy-17β-estradiol (4-MeO-E2), 17-epiestriol (17-epi-E3), 16-epiestriol (16-epi-E3), 16α-hydroxyestrone (16α-OH-E1), 16-keto-17β-estradiol (16-keto-E2), and 2-hydroxyestriol (2-OH-E3), were obtained from Steraloids (Newport, RI, USA). A deuterium-labeled internal standard (IS), 2,4,16,16-*d*_4_-17β-estradiol (*d*_4_-E2, isotopic purity ≥ 98%), was purchased from C/D/N Isotopes (Pointe-Claire, Quebec, Canada).

In solid-phase extraction (SPE), Oasis HLB (3 cc, 60 mg; Waters, Milford, MA, USA) preconditioned with 2 mL of methanol, followed by 2 mL of deionized water was used. Sodium acetate (reagent grade), acetic acid (glacial, 99.99 + %), and l-ascorbic acid (reagent grade) were acquired from Sigma (St. Louis, MO, USA). A solution of β-glucuronidase/arylsulfatase from *Helix pomatia* was purchased from Roche Diagnostics GmbH (Mannheim, Germany). Anhydrous potassium carbonate (K_2_CO_3_), triethylamine (TEA), and ethyl chlorofomate (ECF) were obtained from J. T. Baker (Phillipsburg, NJ, USA), Sigma, and Daejung Chemical Co. (Shiheung, Gyoungi, Korea), respectively. The perfluoroacylation reagent, pentafluoropropionic anhydride (PFPA), was also obtained from Sigma. All organic solvents used were analytical and HPLC-grade and were purchased from Burdick and Jackson (Muskegon, MI, USA). Deionized water was prepared with a Milli-Q purification system (Millipore, Billerica, MA, USA).

### GC-MS-based quantitative estrogen profiling

Quantitative estrogen profiling was performed according to a previous technique [26]. Briefly, the urine sample (2 mL) containing 100 μL of 0.2% aqueous l-ascorbic acid was spiked into 15 μL of the internal standard *d*_4_-E2 (1 μg/mL). The samples were extracted with Oasis HLB SPE cartridges that were placed into a device fitted with a small peristaltic pump that was operated at a flow rate < 1 mL/min. After loading each sample into a cartridge, the cartridges were washed with 2 mL of water and eluted twice with 2 mL of methanol. The combined methanol was evaporated under a nitrogen stream and then added to 1 mL of 0.2 M acetate buffer (pH 5.2), 100 μL of aqueous 0.2% l-ascorbic acid, and 50 μL of β-glucuronidase/arylsulfatase. After a 3-h incubation at 55°C, the solution was adjusted to pH 8 with a 5% K_2_CO_3_ solution, and then 30 μL of TEA and 50 μL of ECF were added. After vortexing for 30 s, the sample was extracted twice with 2.5 mL of a nonpolar solvent (*n*-hexane). The organic solvent was evaporated in an N_2_ evaporator at 40°C, and the sample was dried in a vacuum desiccator over P_2_O_5_-KOH for at least 30 min. Finally, the dried residue was derivatized with 20 μL of PFPA in 100 μL of *n*-hexane at 50°C for 30 min and then evaporated in an N_2_ evaporator. Two microliters of the resulting product, reconstituted with 40 μL of *n*-hexane, were injected for GC-MS analysis with the selected-ion monitoring (SIM) mode.

The GC-SIM/MS was performed on an Agilent 6890 Plus gas chromatograph interfaced with a single-quadrupole Agilent 5975C MSD. The electron energy was 70 eV, and the ion source temperature was 230°C. Each sample (2 μL) was injected in split mode (10:1) at 280°C and separated through an MXT-1 cross-linked dimethylpolysiloxane capillary column (30-m × 0.25-mm I.D., 0.25-μm film thickness, Silcosteel-treated stainless steel). The initial oven temperature was 270°C, which was increased to 300°C at 6°C/min, and finally increased to 330°C with a 10°C/min ramping program. The column head pressure, with helium as the carrier gas, was set to 151.7 kPa. The calibration curve consisted of a blank sample and 11 samples from LOQ. This method was linear, with a correlation coefficient (*r*^2^ > 0.996) for all estrogens analyzed, and the intraday coefficients of variation ranged from 1.3% to 7.7%.

### Statistical analysis

Data manipulation was performed with SigmaPlot (Systat Software Inc., San Jose, CA, USA) and the SPSS (v. 21) for Windows software package (SPSS Inc., Chicago, IL, USA). The concentrations of the individual estrogens and metabolic ratios were calculated by dividing the substrate concentration by that of its metabolite (as an indicator of enzyme activity) in the urine samples obtained from postmenopausal women. Statistical significance was determined with nonparametric Kruskal-Wallis and Mann–Whitney *U* tests to evaluate possible differences in the diagnostic factors. The significant variables with *P* < 0.05 in the Kruskal-Wallis test were then compared with Mann–Whitney *U* tests between 2 groups, including benign versus stage I PTC, benign versus stage III/IV PTC, and stage I versus stage III/IV PTC. A Bonferroni correction was used to adjust for the 2 comparisons performed on each variable and to set the significance level at 0.017. The concentration of estrogens was listed in Supplementary Data as an Additional file 1.

## Results

### Urinary concentrations of estrogens in patients with benign and malignant tumors

The individual urinary estrogen levels in the postmenopausal women with benign and malignant tumors were evaluated. Among the 16 estrogens monitored, 15 estrogens, including parent estrogens (E1 and E2), 16α-hydroxylation metabolites (16α-OH-E1, 16-keto-E2, E3 [16α-OH-E2], 17-epi-E3, and 16-epi-E3), 2- and 4-hydroxylation metabolites (2-OH-E1, 2-OH-E2, 4-OH-E1, and 4-OH-E2), and 2- and 4-methoxylation metabolites (2-MeO-E1, 2-MeO-E2, 4-MeO-E1, and 4-MeO-E2), were quantitatively detected (Table 1). No 2-OH-E3 was detected in this study.

**Table 1:** Urinary estrogen concentrations associated with benign tumors and malignant Papillary Thyroid Carcinomas (PTCs) in the postmenopausal women

**Compounds (trivial name)**	**Benign tumor (a) (*n*** **= 9)**	**Malignant PTCs**			***P*-value**^**2**^		
		**Stage I (b) (*n*** **= 11)**	**Stage III/IV (C) (*n*** **= 7)**	***P*-value**^**1**^	***a *** **vs. *b***	***a *** **vs. *c***	***b *** **vs. *c***
Estrone (E1)	12.1 ± 11.3	2.9 ± 2.3	5.0 ± 6.5	0.053	0.020	0.091	0.791
17β-estradiol (E2)	0.8 ± 0.6	0.4 ± 0.2	1.1 ± 1.3	0.416			
Estriol (E3)	1.7 ± 1.7	1.9 ± 1.7	6.2 ± 6.7	0.146			
2-hydroxyestrone (2-OH-E1)	2.2 ± 3.5	0.3 ± 0.3	0.7 ± 1.2	0.073			
2-hydroxy-17β-estradiol (2-OH-E2)	0.5 ± 0.4	0.1 ± 0.1	0.3 ± 0.2	0.052	0.031	0.536	0.085
4-hydroxyestrone (4-OH-E1)	0.6 ± 0.5	0.3 ± 0.3	0.4 ± 0.4	0.558			
4-hydroxy-17β-estradiol (4-OH-E2)	0.5 ± 0.4	0.1 ± 0.1	0.2 ± 0.1	0.182			
2-methoxyestrone (2-MeO-E1)	1.6 ± 1.7	0.5 ± 0.4	0.6 ± 0.6	0.108			
2-methoxy-17β-estradiol (2-MeO-E2)	1.4 ± 1.1	0.4 ± 0.3	0.6 ± 0.4	0.034	0.012	0.114	0.285
4-methoxyestrone (4-MeO-E1)	0.6 ± 0.8	0.1 ± 0.1	0.2 ± 0.4	0.011	0.004	0.174	0.179
4-methoxy-17β-estradiol (4-MeO-E2)	0.3 ± 0.7	0.1 ± 0.1	0.2 ± 0.2	0.104			
17-epiestriol (17-epi-E3)	0.3 ± 0.3	0.1 ± 0.1	0.1 ± 0.1	0.029	0.016	0.142	0.425
16-epiestriol (16-epi-E3)	0.7 ± 0.9	0.3 ± 0.2	0.9 ± 1.0	0.589			
16α-hydroxyestrone (16α-OH-E1)	0.9 ± 0.7	0.6 ± 0.6	0.8 ± 0.8	0.462			
16-keto-17β-estradiol (16-keto-E2)	0.7 ± 0.9	0.6 ± 0.6	0.7 ± 1.0	0.803			

Concentrations are expressed as ng/mg of creatinine (mean ± SD).

Statistical significance was determined by a nonparametric ^1^Kruskal-Wallis and ^2^Mann-Whitney *U* test to evaluate possible differences in the diagnostic factors.

The significant estrogens with *P* < 0.05 in a Kruskal-Wallis test were then compared with the Mann–Whitney *U* test results between 2 groups, including benign versus stage I PTC (a vs. b), benign versus stage III/IV PTC (a vs. c), and stage I versus stage III/IV PTC (b vs. c). A Bonferroni correction was applied to the post hoc analysis of the between-group comparisons performed for each variable and to set the significance level at 0.017.

To characterize the differential excretion patterns of postmenopausal malignant thyroid carcinoma patients according to the tumor stage, samples were analyzed from the patients diagnosed at early (stage I) and advanced (stage III/IV) stages, and the samples from each group were compared with the control benign samples. Differences in the concentrations of 2-MeO-E2, 4-MeO-E1, and 17-epi-E3 were statistically significant between the benign and stage I malignant tumors. The urinary levels of these substances were found to be significantly reduced in stage I malignant PTCs (*P* < 0.02); in particular, the levels of 2-MeO-E2 and 4-MeO-E1 in early-stage malignant patients were >3.5-fold (*P* = 0.012) and >6.0-fold (*P* = 0.004) lower than those in the benign samples. There were no statistically significant differences between the malignant PTCs stages.

### Metabolic alterations with benign tumors and malignant PTCs

**Figure 1:**
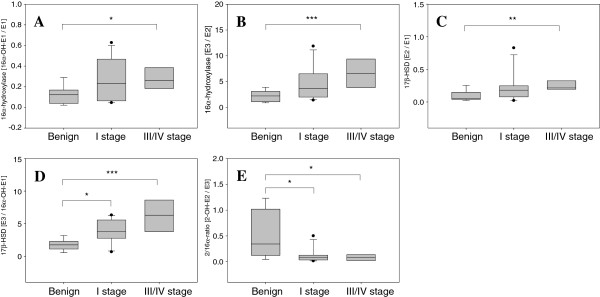
**Differential metabolic ratios on estrogen metabolism with statistical significance in benign and malignant papillary thyroid tumors.** Estrogen activities were compared in postmenopausal women with benign adenomas (*n* = 9) and stage I (*n* = 11) and III/IV (*n* = 7) malignant carcinomas. The significant metabolic ratios with *P* < 0.05 in a Kruskal-Wallis test were analyzed with the Mann–Whitney *U* test between 2 groups, including benign versus stage I PTC, benign versus stage III/IV PTC, and stage I versus stage III/IV PTC. **(A)** 16α-OH-E1/E1, **(B)** E3/E2, **(C)** E2/E1, **(D)** E3/16α-OH-E1 and **(E)** 2-OH-E2/E3. * *P* < 0.02; ** *P* < 0.002; and *** *P* < 0.0002.

Based on the individual concentrations, the metabolite to precursor ratios, which could be used to gain insight into metabolic enzyme activity, were examined to understand the alterations to estrogen metabolism with respect to thyroid tumors (Table 2). In contrast to the individual concentrations, significant changes between the patients with benign tumors and those with malignant PTCs, with either stage I or III/IV diagnosis, were confirmed by the differences in these metabolic ratios. For 16α-hydroxylase, the ratios of 16α-OH-E1 to E1 (Figure 1A) and E3 to E2 (Figure 1B) were significantly higher in patients with stage III/IV malignant PTCs than in those with benign tumors (*P* < 0.02 each). There were no significant changes in the metabolic activity of 4-hydroxylase of estrogens and catechol-*O*-methyltransferase of 2 and 4-hydroxy estrogens.

**Table 2:** Metabolic ratios of urinary estrogens between benign tumors and malignant Papillary Thyroid Carcinomas (PTCs) in postmenopausal women

**Metabolic ratio (product/precursor)**	**Benign tumor (a) (*n*** **= 9)**	**Malignant PTCs**			***P*-value**^**2**^		
		**Stage I (b)**	**Stage III/IV (c)**	***P*-value**^**1**^	***a *** **vs. *b***	***a *** **vs. *c***	***b *** **vs. *c***
		**(*n*** **= 11)**	**(*n*** **= 7)**				
**2-hydroxylase**							
2-OH-E1/E1	0.16 ± 0.14	0.13 ± 0.09	0.16 ± 0.15	0.991			
2-OH-E2/E2	0.75 ± 0.40	0.38 ± 0.30	0.47 ± 0.29	0.070			
**4-hydroxylase**							
4-OH-E1/E1	0.10 ± 0.13	0.12 ± 0.09	0.15 ± 0.08	0.182			
4-OH-E2/E2	0.64 ± 0.44	0.42 ± 0.28	0.40 ± 0.20	0.360			
**16α-hydroxylase**							
16α-OH-E1/E1	0.12 ± 0.09	0.26 ± 0.20	0.31 ± 0.20	0.052	0.095	0.012	0.659
E3/E2	2.16 ± 1.10	4.72 ± 3.28	6.66 ± 3.27	0.007	0.067	0.001	0.126
**Methyltransferase**							
2-MeO-E1/2-OH-E1	1.43 ± 0.94	1.68 ± 1.00	1.66 ± 0.93	0.838			
2-MeO-E2/2-OH-E2	6.35 ± 8.80	4.98 ± 5.47	3.55 ± 4.53	0.935			
4-MeO-E1/4-OH-E1	3.23 ± 6.88	0.25 ± 0.18	0.58 ± 0.47	0.140			
4-MeO-E2/4-OH-E2	4.02 ± 7.28	0.83 ± 0.57	1.20 ± 0.60	0.216			
**17β-hydroxysteroid dehydrogenase**							
E2/E1	0.10 ± 0.08	0.23 ± 0.22	0.26 ± 0.08	0.013	0.046	0.002	0.285
E3/16α-OH-E1	1.75 ± 0.80	3.79 ± 1.80	6.15 ± 2.18	0.001	0.010	0.0001	0.044
**2-/16α-hydroxylation**							
2-OH-E1/16α-OH-E1	2.05 ± 2.46	0.82 ± 0.74	0.64 ± 0.58	0.110			
2-OH-E2/E3	0.50 ± 0.46	0.11 ± 0.14	0.08 ± 0.06	0.022	0.020	0.012	0.930

The metabolic ratios are presented as the metabolite-to-precursor ratio in each subject (mean ± SD).

Statistical significance was determined with nonparametric ^1^Kruskal-Wallis and ^2^Mann-Whitney *U* tests to evaluate possible differences in the diagnostic factors.

The significant metabolic ratios with *P* < 0.05 in the Kruskal-Wallis test were then compared with the Mann–Whitney *U* test between 2 groups, including benign versus stage I PTC (a vs. b), benign versus stage III/IV PTC (a vs. c), and stage I versus stage III/IV PTC (b vs. c). A Bonferroni correction was applied to the post hoc analysis of the between-group comparisons performed for each variable and to set the significance level at 0.017.

Reductive 17β-hydroxysteroid dehydrogenase (17β-HSD), which converts E1 into E2 and 16α-OH-E1 into E3 by reduction at the C17 position [19], was significantly different in samples from patients with malignant PTCs and those from patients with benign tumors (Figure 1C-D). The ratios of E2 to E1 (*P* = 0.046 for benign vs. stage I; *P* = 0.002 for benign vs. stage III/IV, Figure 1C) and of E3 to 16α-OH-E1 (*P* = 0.010 for benign vs. stage I; *P* = 0.0001 for benign vs. stage III/IV, Figure 1D) could differentiate benign from malignant tumor samples. In addition, a significant reduction in urinary 2-/16α-hydroxylation pathway was observed in patients with stage III/IV malignant PTC, compared with those with benign tumors (>6.2-fold difference; *P* = 0.012; Figure 1E).

## Discussion

Over the last decades, many studies based on gene or protein expression *in vitro* and *in vivo* have evaluated the role of biomarkers in determinations of thyroid tumor malignancy [6-10], but candidate biomarkers such as galectin-3 or MET have not shown the sensitivity and specificity needed for a preoperative thyroid nodules screening tool [28]. We previously evaluated metabolic changes in urinary steroid levels in premenopausal and postmenopausal women and men with PTCs by quantitative steroid profiling, as PTC pathogenesis and development might be affected by androgens and estrogens [14]. However, these results did not reveal information about the changes in estrogen metabolism that could distinguish PTCs from benign thyroid tumors. Therefore, this study focused more on the metabolic differences between benign tumors and early and advanced-stage malignant PTCs in postmenopausal women.

The 2-MeO-E2 levels significantly decreased in both early and advanced PTCs, suggesting that this could serve as a potential biomarker to differentiate benign from malignant thyroid neoplasms. 2-MeO-E2, which acts as a potential antitumor agent in several types of cancers [29,30], blocks cell growth and induces apoptosis in thyroid carcinoma cells by activating the p38 mitogen-activated protein kinase [31]. Based on its cellular effects, 2-MeO-E2 might be expected to attenuate the progression of tumorigenesis and protect against the development of malignant PTCs from benign adenomas.

Estrogen metabolism was shown to potentiate the growth of several cancers, including breast, prostate, endometrial, and thyroid cancers [12-14,20-23,25,31]. For E2, the C-2/C-16α-hydroxylation ratio and level of 16α-hydroxylation could be used as predictive biomarkers for breast cancer [20-22], suggesting that the major estrogen metabolism pathways have different biological actions such as the antiproliferative effect of 2-hydroxylation and proliferative effect of 16α-hydroxylation in hormone-related cancers [12,18-23]. In this study, 16α-hydroxylation of both E1 and E2 was markedly increased in both early and advanced-stage malignant PTCs. In particular, the influence of estrogen 16α-hydroxylation tended to be in clear contrast to that of 2-hydroxylation in postmenopausal low- and high-grade PTCs (Table 2).

Increased 16α-hydroxylation activity preceded clinical evidence of cancer and therefore might represent a significant risk factor for estrogen-dependent tumor development [20-23]. In this study, the 2-OH-E1-to-16α-OH-E1 ratio was not statistically significantly different between the malignant PTC and benign groups, despite the lower activity in the former group. However, a decreased 2-OH-E2-to-E3 ratio was observed. For E2, the ratio of C-2 and C-16α hydroxylation in the malignant PTC groups might reflect the observed effects of increased 16α-hydroxylation on malignant PTC in postmenopausal women. This observation is consistent with previous studies that examined the extent of the interplay between 2-hydroxylation and 16α-hydroxylation and reflected that the estrogen metabolite ratio could be a distinguishing feature between benign and malignant thyroid tumors [12].

Another interesting estrogen profile pattern was detected between the benign and malignant tumors. The expression of reductive 17β-HSD in patients with both early and late-stage PTCs was significantly higher than that in the benign patients; this affected the conversions of E1 to E2 and 16α-OH-E1 to E3 (Table 2). Although the underlying mechanism is not clear, the E3-to-16α-OH-E1 ratio progressively increased from the benign group to the early stage malignant PTC group and finally to the advanced-stage malignant PTC group, and had a more significant *P* value than the E2 to E1 ratio. In recent, the implications of 17β-HSD activity in tumorigenesis have been investigated [32-34]. In these studies, high 17β-HSD activity was related to increased cell proliferation, and it was important to the understanding of the mechanisms that underlie breast cancer development [32,33]. Reductive 17β-HSD is also overexpressed in endometrial cancer, compared with control tissues, and was found to increase tumor cell proliferation [34]. To our knowledge, this is the first study to demonstrate an association between 17β-HSD expression and pathological features in patients with PTCs.

This study had 2 limitations. First, a small number of postmenopausal women with thyroid disease (only 27 individuals) limited the statistical power with which to distinguish between benign and malignant tumors. Despite these drawbacks, however, very few fundamental studies on this topic have been performed in postmenopausal women. Although we could not conclusively determine the associations between estrogen metabolism and pathological features in patients with PTCs, we have demonstrated statistically significant estrogen metabolic patterns and differentiated metabolic pathways. Second, high variability in the urinary estrogen levels was detected in all postmenopausal patients, including those of advanced age. Most circulating estrogens in elderly women are derived from adipose tissues and can be affected by additional factors such as nutrition, smoking, alcohol intake, and physical activity [35]. There is considerable intra- and inter-patient estrogen variability among postmenopausal women in response to estrogen replacement therapy [36]. In contrast to the metabolic ratios, the urinary levels of the individual estrogens, except 2-MeO-E2, were not significantly different between benign and malignant thyroid tumor patients because of the high variability in the urinary estrogen levels. Further studies with a large population of individuals with thyroid tumors are needed to better define the changes in individual estrogens in PTC patients.

## Conclusions

Malignant PTCs can be distinguished from benign thyroid nodules by urinary estrogen profiling. These observations suggest that activation of the 16α-hydroxylation pathway and reductive 17β-HSD expression are important endocrine factors in thyroid tumorigenesis, similar to their roles in other endocrine-related cancers such as breast and endometrial cancers. These results could provide insights into the pathogenesis of PTC and might ultimately describe a useful potent biomarker for prognostic evaluations of malignant PTC in clinical practice and for therapeutic management in patients with thyroid tumors.

## Competing interests

The authors declare that they have no competing interests.

## Authors’ contributions

JYM prepared the manuscript and conducted the estrogen profiling. EJL and WYC participated in preparing the experimental protocol, clinical diagnosis, and sampling. MHM and BCC participated in the study design and helped to draft the manuscript. MHC conceived of the study and participated in its design and coordination. All the authors revised the manuscript and approved the final version.

## Pre-publication history

The pre-publication history for this paper can be accessed here:

http://www.biomedcentral.com/1472-6890/13/25/prepub

## Supplementary Material

Additional file 1 Individual concentration of estrogens studied (ng/mg of creatinine). Click here for file
